# Supplementary material for: Symmetry Breaking in Chemical Systems: Engineering Complexity Through Self‐Organization and Marangoni Flows
Source: Adv Sci (Weinh). 2025 Oct 17;13(5):e15672. doi: 10.1002/advs.202515672 (PMC12850104; doi:10.1002/advs.202515672)
Supplement: Supplementary file 1 — Supporting Information [file ADVS-13-e15672-s001.pdf]

Supplemental Information-Symmetry Breaking in Chemical Systems: Engineering Complexity through Self-Organization and Marangoni Flows

*Sangram Gore, Binaya Paudyal, Duarte Rocha, Mohamed Ali, Nader Masmoudi, Albert Bae, Christian Diddens, Detlef Lohse, Oliver Steinbock, Azam Gholami**

Dr. S. Gore, B. Paudyal, Dr. M. Ali, Prof. N. Masmoudi, Prof. A. Gholami
Science Division, New York University Abu Dhabi, Abu Dhabi, UAE
Email Address: azam.gholami@nyu.edu
D. Rocha, Dr. C. Diddens, Prof. D. Lohse
Physics of Fluids Department, Max-Planck Center Twente for Complex Fluid Dynamics and J. M. Burgers Centre for Fluid Dynamics, University of Twente, Enschede, The Netherlands
Prof. D. Lohse
Max-Planck Institute for Dynamics and Self-Organization, Am Faßberg 17, 37077 Göttingen, Germany
Prof. N. Masmoudi
Courant Institute of Mathematical Sciences, New York University, New York, USA
Prof. A. Bae
Lewis & Clark College, Portland, Oregon, USA
Prof. O. Steinbock
Department of Chemistry and Biochemistry, Florida State University, Tallahassee, Florida, USA

Keywords: *Belousov-Zhabotinsky reaction, Marangoni flows, Chemo-hydrodynamic patterns, Hydrodynamic fingering instabilty, Reaction-diffusion systems*

S1 Supplemental Figures

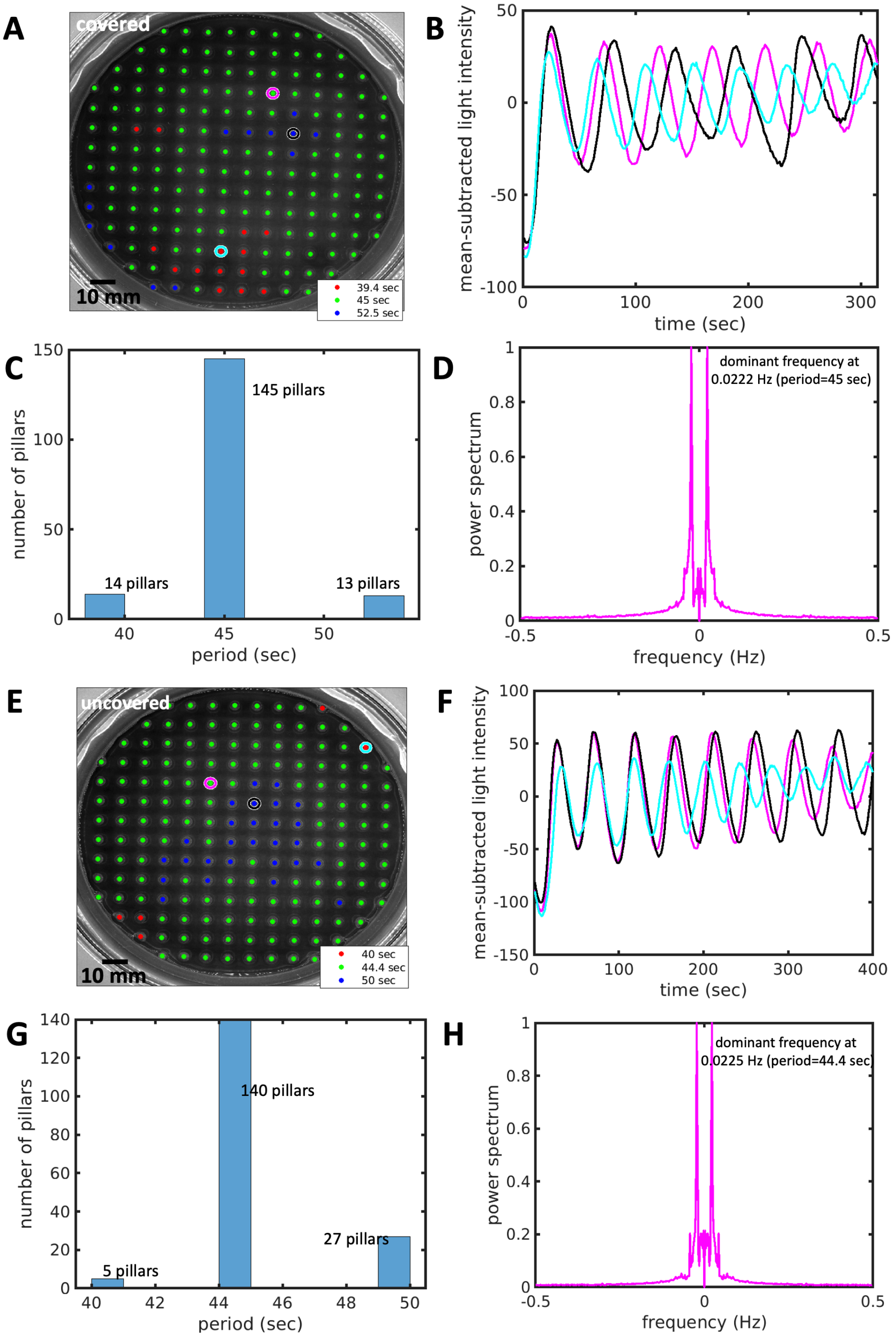

Figure S1: **Frequency Analysis**. (**A**) Color-coded period of the waves centered around the obstacles, measured in seconds. The Petri dish is covered and the waves maintain their circular shapes over multiple cycles, as shown in Figure 1K. (**B**) Averaged light intensity oscillations around three selected pillars highlighted in panel A, showing oscillations with periods of 45 seconds (magenta), 52.5 seconds (black), and 39.4 seconds (cyan). (**C**) Histogram of oscillations period. (**D**) Power spectrum of the oscillations shown in magenta in panel (B). (**E-H**) Similar to panels (A-D), but with the Petri dish uncovered, affecting the wave dynamics as shown in Figure 1L.

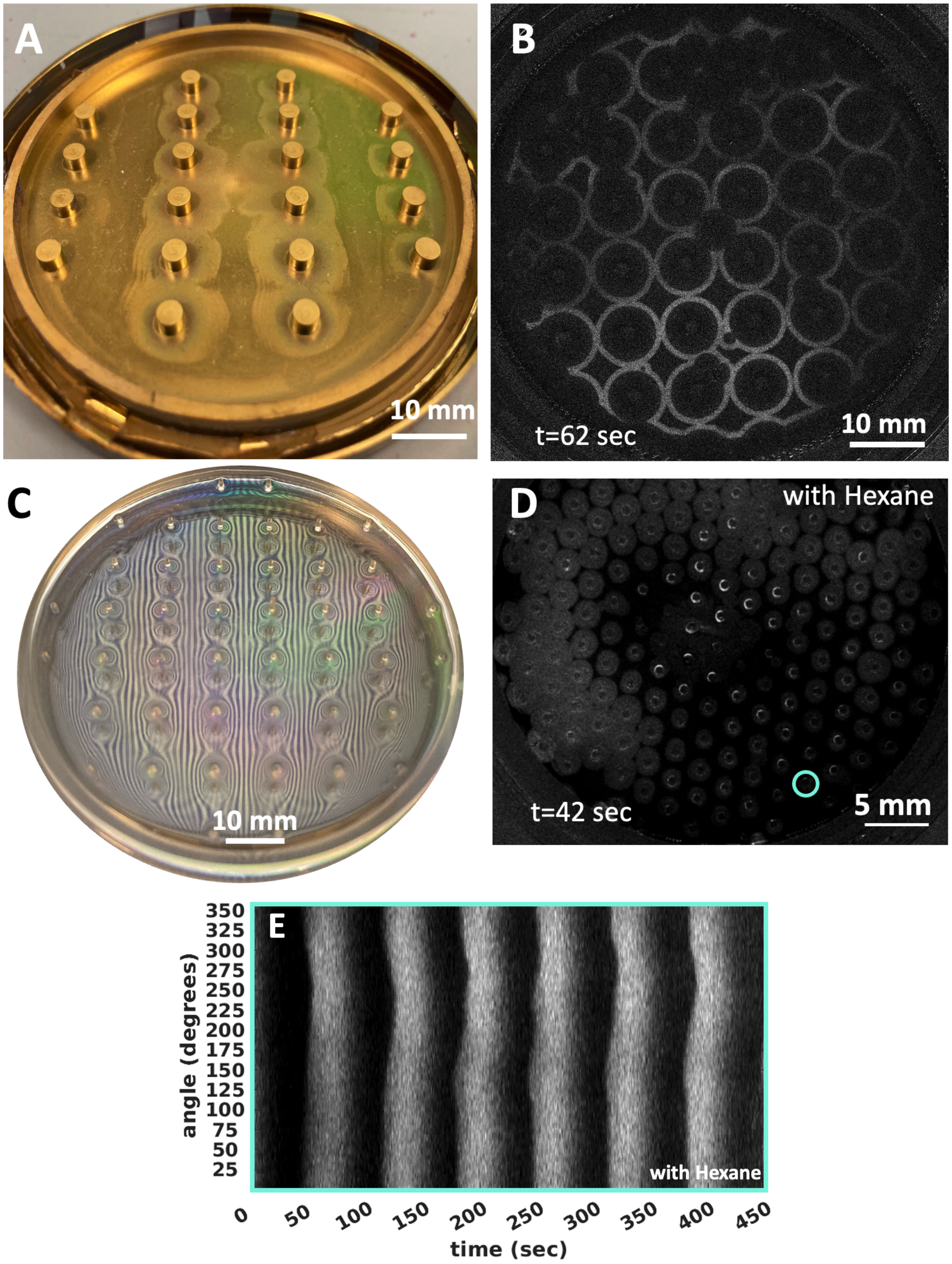

Figure S2: (**A**) A sample of a PDMS mold coated with a thin layer of gold is shown. The large pillars have a diameter of 3.8 mm. (**B**) Synchronous waves are centered around the gold-coated pillars. (**C**) A striped pattern is projected onto the fluid surface, highlighting the deformation caused by the rising fluid around the hydrophilic pillars. **(D)** A thin layer of hexane (with about 1 mm thickness) over the CHD-BZ solution prevents evaporation, ensuring the wavefronts preserve their circular shapes. Please note that the obstacles are not submerged. **(E)** Space-time plot along the cyan circle in panel D illustrating that over time the wavefronts maintain their circular shape.

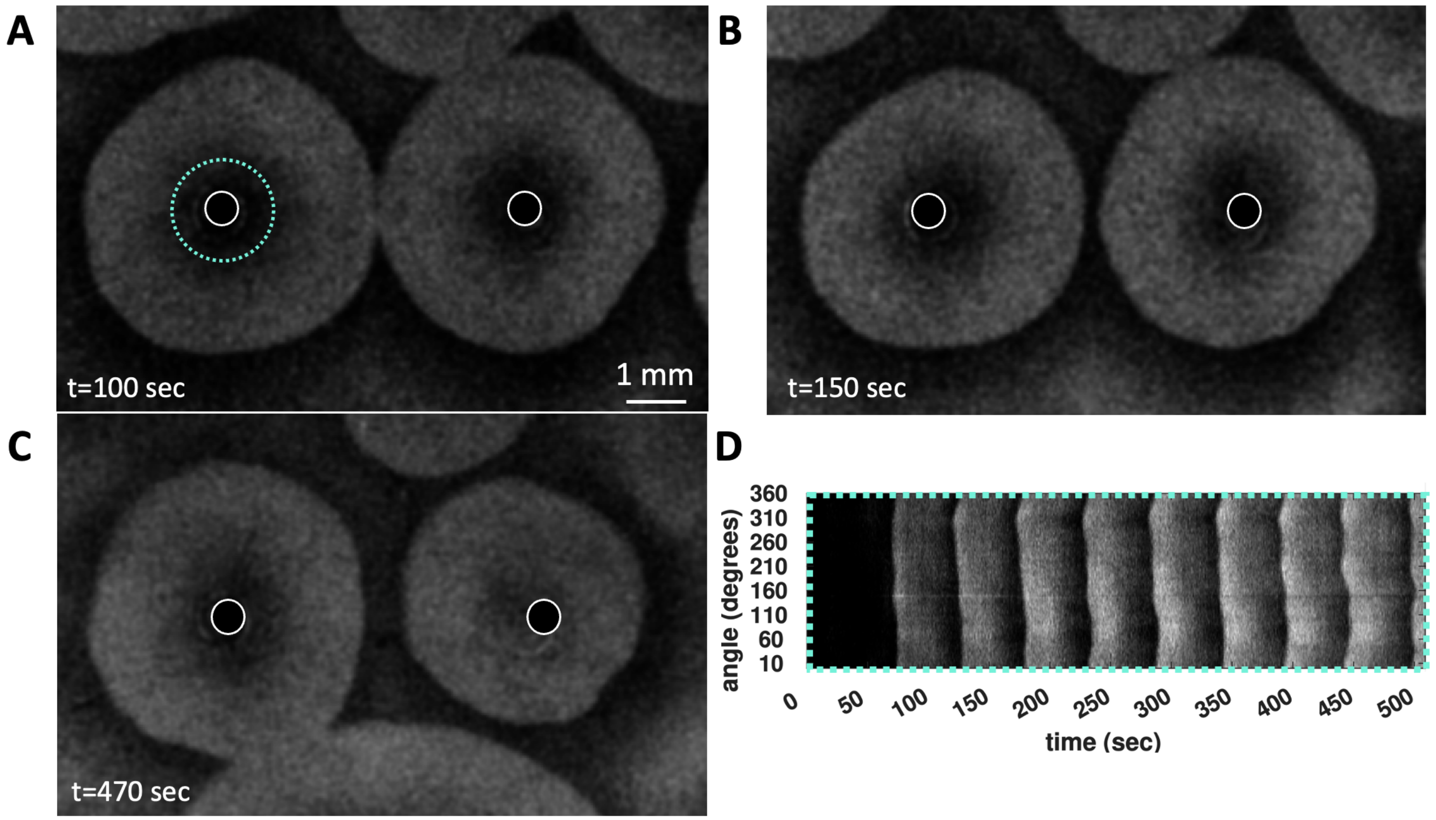

Figure S3: **No fingering instability in small-diameter pillars**. (**A**-**D**) In experiments conducted with uncovered Petri dishes featuring pillars of 0.5 mm diameter, there is no occurrence of wavefront instability. This observation indicates that a certain minimum perimeter of the pillars is necessary for fingering instability to occur. The space-time plot shown in panel (D) is created by accumulating the light intensity around the cyan-colored circle depicted in panel (A).

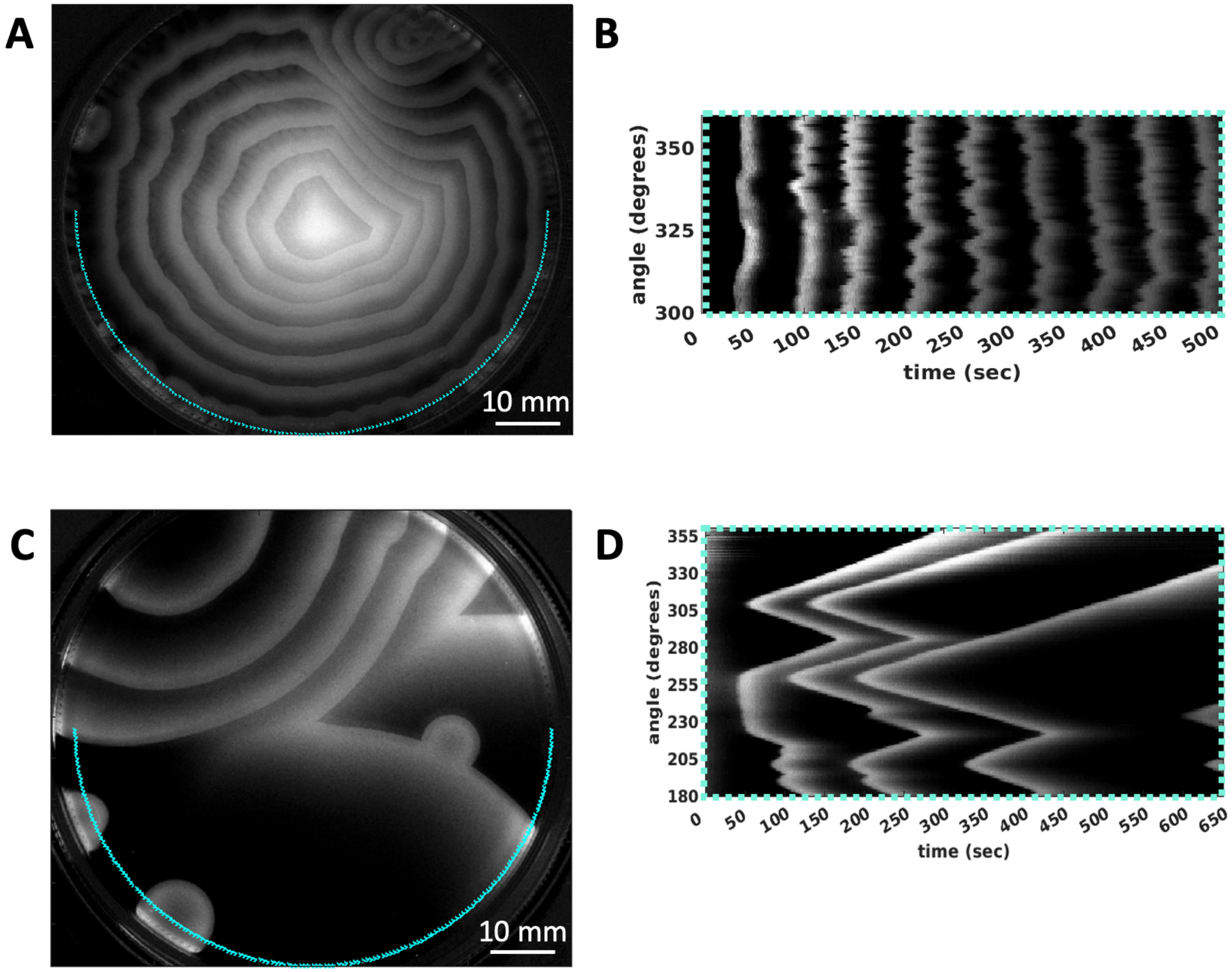

Figure S4: **Experiment in a glass Petri dish without obstacles**. (**A**) A top view of a glass uncovered Petri dish with plasma treatment, illustrating fingering instability at the glass boundary. Notice that rippled waves travel and make visible the underlying mosaic patterns generated by the Marangoni instabilities. (**B**) A space-time plot along the cyan-colored semicircle shows the dynamics of the fingering, emphasizing that the positions of the fingers can change over time. (**C-D**) A setup similar to panel (A), but covered to minimize evaporation. In this configuration, wave centers appear at the periphery, but the wavefronts maintain their circular forms.

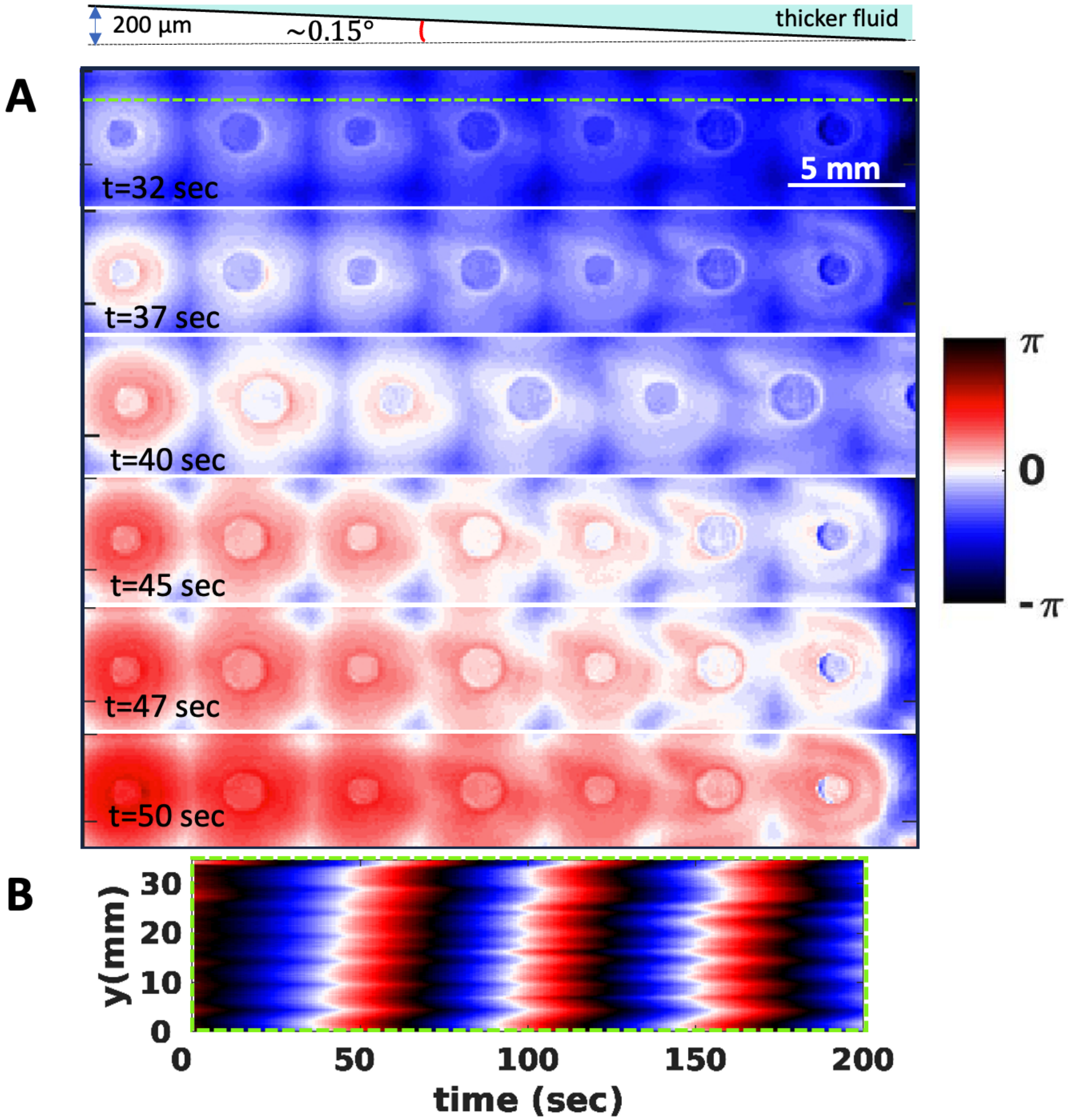

Figure S5: **Phase map of an experiment in a tilted Petri dish** (**A**) In a Petri dish with a slight tilt, synchronization waves initiate in the thinner part of the fluid and propagate towards the area with greater fluid thickness (video 15). (**B**) Space-time plot of the phase map along the green dashed line indicated in panel (A) illustrates the propagation of phase waves. Notice that in this experiment, the Petri dish is not covered, and flower patterns emerge at $t \sim 80$ sec.

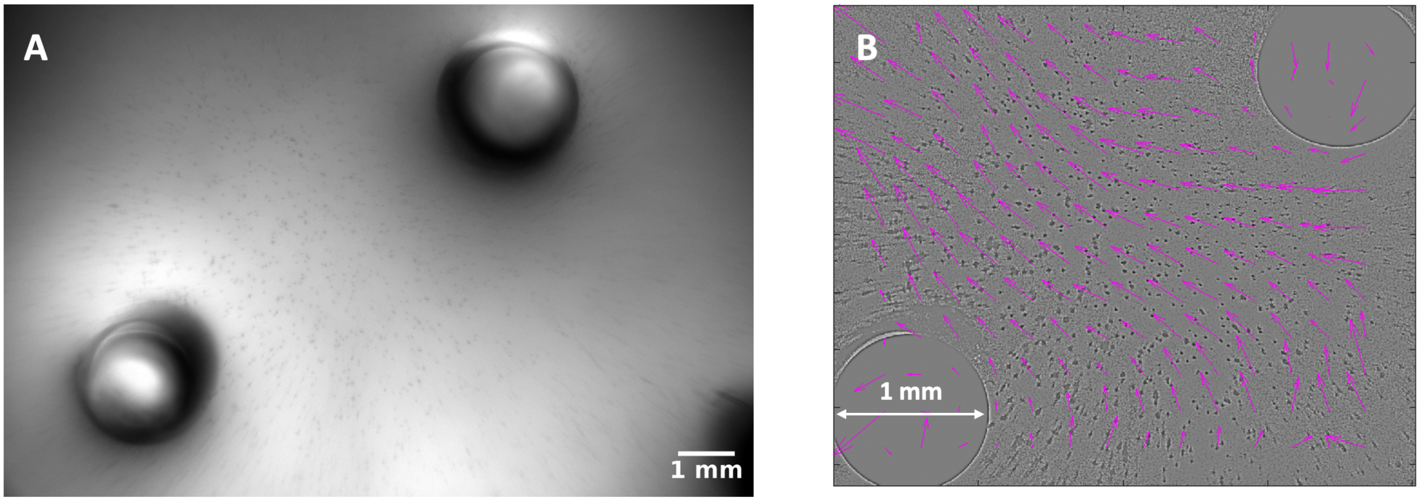

Figure S6: **Marangoni-driven surface flows.** (**A-B**) In this experiment, tracer particles (20 μm in diameter) are used to visualize surface flows driven by Marangoni flows. These flows arise from chemical concentration gradients and slight temperature differences caused by evaporative cooling, which generate surface tension gradients (refer to video 27).

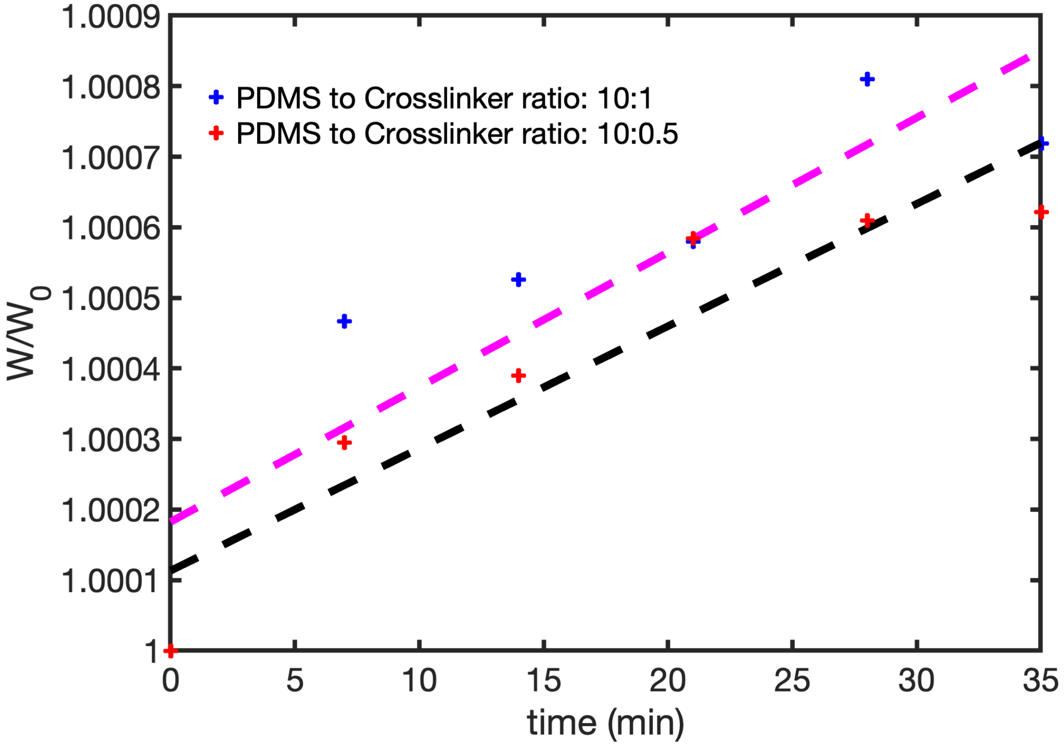

Figure S7: **Negligible swelling of PDMS:** To rule out PDMS swelling during our experiments, we measured the weight of the PDMS after each recording and found negligible changes. The initial PDMS weight at time zero is denoted as W_0. Data are presented for different PDMS-to-cross-linker ratios. All experiments were conducted using a PDMS-to-cross-linker ratio of 10:1. Even with a softer PDMS ratio of 10:0.5, the swelling effect remained negligible.

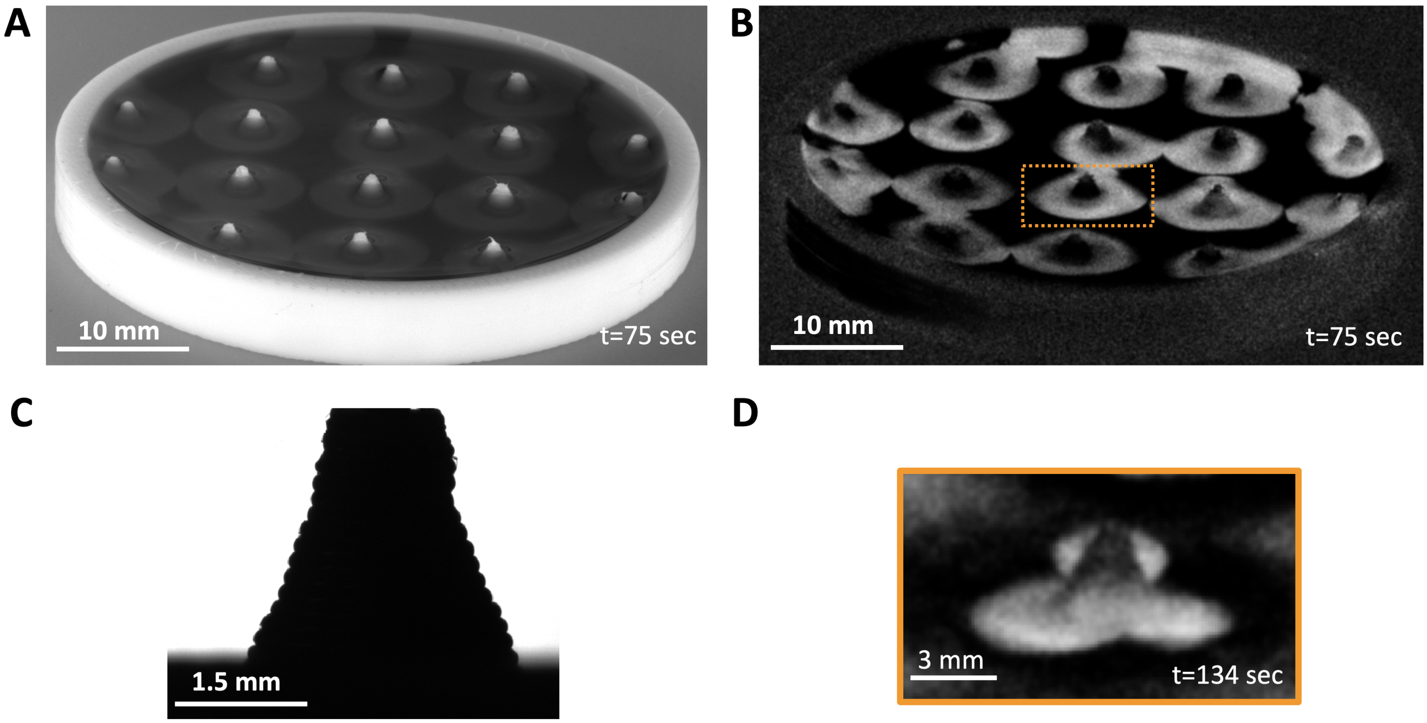

Figure S8: **Experiments with rough obstacles:** To examine the impact of surface roughness, we used a conical array of 3D-printed PLA (polylactic acid) obstacles with a wavy surface. Despite their rough texture, these obstacles continued to act as wave centers. In an open setup, the circular wavefronts fragmented, forming flower-like patterns, though with greater variability in petal size. The image in panel B is a processed version of the image in panel A, where the temporal mean has been subtracted, and a Gaussian blur has been applied.

S2 Meniscus analysis

At static equilibrium, the pressure jump across a liquid–air interface equals surface tension times mean curvature (Young–Laplace law):

$$\gamma\,\kappa \;=\; \Delta P. \tag{1}$$

Measuring height $H(r)$ upward (relative to a far-field reference) gives the hydrostatic pressure difference $\Delta p = \rho g\,H$, where ρ is the liquid–air density difference and g is gravity. For an axisymmetric meniscus $z \;=\; H(r)$ around a vertical cylinder, the exact mean curvature is the sum of principal curvatures:

$$\kappa \;=\; \frac{H''}{\left(1+H'^2\right)^{3/2}} \;+\; \frac{1}{r}\,\frac{H'}{\sqrt{1+H'^2}}, \tag{2}$$

where primes denote derivatives with respect to r.
Let R be the cylinder radius and define nondimensional variables $\tilde{r} \;=\; r/R$ and $\tilde{H} \;=\; H/R$. Combining (1)–(2) yields the exact (nonlinear) shape equation

$$\frac{d}{d\tilde{r}}\left[\frac{\tilde{r}\,\tilde{H}'}{\sqrt{1+(\tilde{H}')^2}}\right] \;-\; \mathrm{Bo}\,\tilde{r}\,\tilde{H} \;=\; 0, \qquad \mathrm{Bo} \equiv \frac{\rho g R^2}{\gamma}, \tag{3}$$

with boundary conditions

$$\tilde{H}'(1) \;=\; -\cot\theta, \qquad \tilde{H}(\tilde{r}) \to 0 \quad \text{as } \tilde{r} \to \infty, \tag{4}$$

where θ is the (macroscopic) contact angle at the wall. The Bond number Bo measures gravity vs. surface tension, and is related to the capillary length $\ell_c = \sqrt{\gamma/(\rho g)}$ via

$$\mathrm{Bo} \;=\; \left(\frac{R}{\ell_c}\right)^2. \tag{5}$$

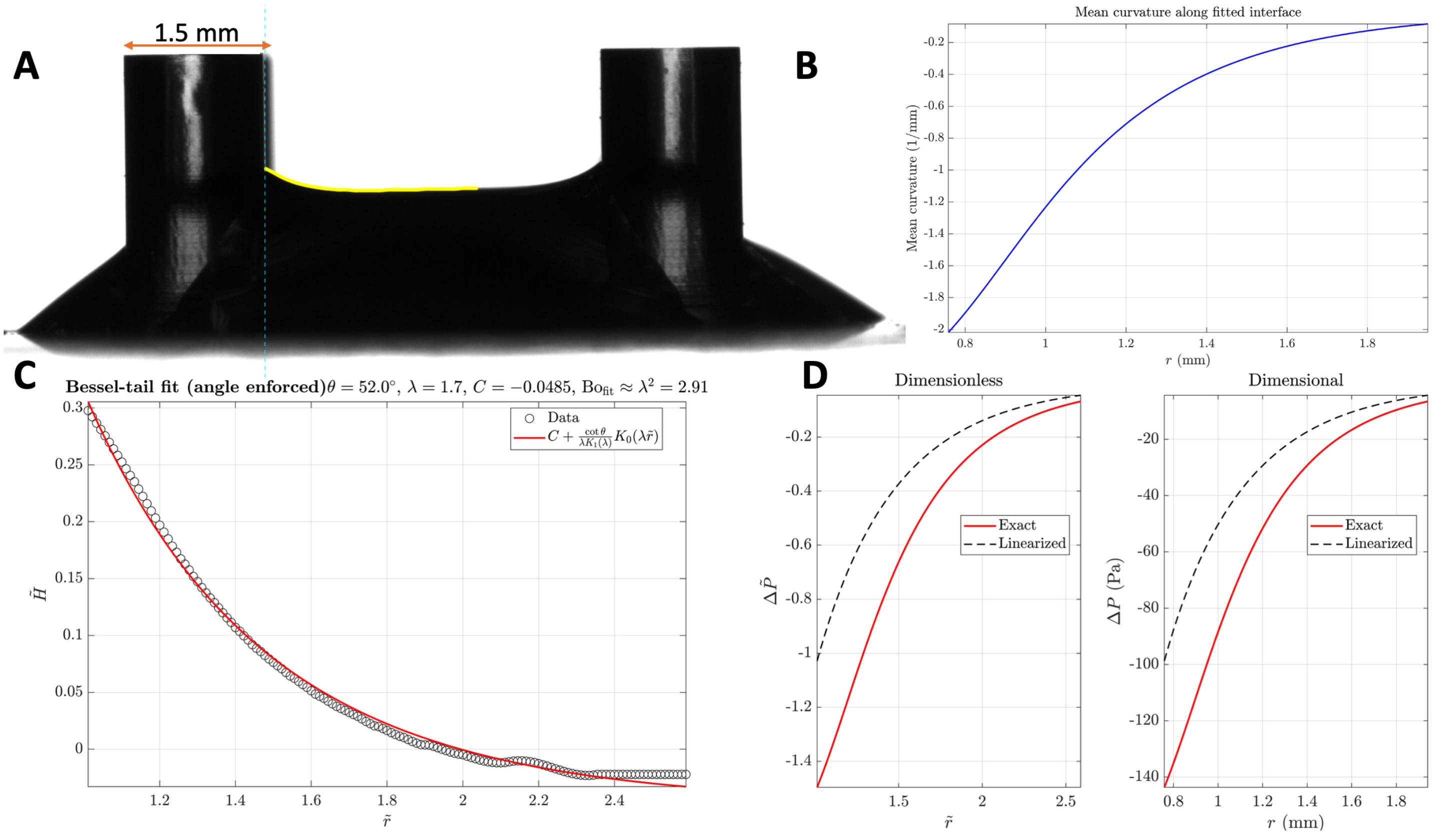

Figure S9: **Meniscus extraction, model fit, and pressure reconstruction.** (A) Side-view of the rising film around a cylindrical obstacle; the interface (yellow) is obtained by edge tracing within a user-defined region of interest, and the dashed line marks the cylinder wall. (B) Dimensionless meniscus profile (circles) together with the best-fit far-field model that enforces the wall contact angle; the example fit returns lambda 1.7, offset -0.0485, and a fitted Bond number of 2.91. (C) Laplace pressure reconstructed along the interface, shown in both dimensionless and dimensional units; the solid red curve uses the exact curvature while the dashed black curve shows a linearized approximation, with the largest differences near the wall.

Far from the wall the slope is small, $|\tilde{H}'| \ll 1$, and the curvature linearizes to $\kappa \approx -(\tilde{H}'' + \tilde{H}'/\tilde{r})$. Equation (3) then reduces to the modified Bessel equation

$$\tilde{H}'' + \frac{1}{\tilde{r}}\,\tilde{H}' - \mathrm{Bo}\,\tilde{H} = 0, \tag{6}$$

whose decaying solution is

$$\tilde{H}(\tilde{r}) \;\propto\; K_0\big(\lambda\,\tilde{r}\big), \qquad \lambda \equiv \sqrt{\mathrm{Bo}} \;=\; \frac{R}{\ell_c}. \tag{7}$$

Asymptotically, $K_0(\lambda\tilde{r}) \sim \sqrt{\pi/(2\lambda\tilde{r})}\, e^{-\lambda\tilde{r}}$, i.e. an exponential decay with a $1/\sqrt{\tilde{r}}$ prefactor. Thus, when lengths are scaled by R, the inverse decay length measured from the meniscus tail is precisely $\lambda = \sqrt{\mathrm{Bo}}$.
To impose the wall boundary condition $\tilde{H}'(1) = -\cot\theta$ while retaining the linear Bessel form in the tail, we use

$$\tilde{H}(\tilde{r}) \;=\; C \;+\; \frac{\cot\theta}{\lambda\,K_1(\lambda)}\,K_0\big(\lambda\,\tilde{r}\big), \tag{8}$$

where C is a baseline offset that absorbs small imaging biases in the far field. Using $dK_0(x)/dx = -K_1(x)$, (8) yields

$$\tilde{H}'(1) = \frac{\cot\theta}{\lambda K_1(\lambda)}\big(-\lambda K_1(\lambda)\big) = -\cot\theta, \tag{9}$$

as required. The fit therefore adjusts only $\lambda > 0$ and C; the amplitude is fixed by the angle constraint. As soon as λ is known, the fitted Bond number follows as

$$\mathrm{Bo}_{\mathrm{fit}} = \lambda^2\,, \qquad \ell_{c,\mathrm{fit}} = \frac{R}{\lambda}\,. \tag{10}$$

Laplace pressure from the fitted shape

The fitted profile can be used to compute the Laplace pressure jump ΔP along the interface. Two complementary approaches are used.
Linearized pressure. In the linear regime, $\kappa_{\text{lin}} \approx -(\tilde{H}'' + \tilde{H}'/\tilde{r})$ and, using (6), one obtains

$$\Delta\tilde{P}_{\text{lin}}(\tilde{r}) \;\equiv\; \kappa_{\text{lin}}(\tilde{r}) \;\approx\; -\,\mathrm{Bo}_{\text{fit}}\left(\tilde{H}(\tilde{r}) - C\right). \tag{11}$$

The dimensional pressure follows from $\Delta P_{\text{lin}} = (\gamma/R)\,\Delta\tilde{P}_{\text{lin}}$.
Exact pressure (nonlinear curvature). Using the analytic derivatives of (8),

$$\tilde{H}'(\tilde{r}) \;=\; -\frac{\cot\theta}{K_1(\lambda)}\,K_1\!\left(\lambda\tilde{r}\right), \qquad \tilde{H}''(\tilde{r}) \;=\; \frac{\cot\theta\,\lambda}{2K_1(\lambda)}\Big[\,K_0\!\left(\lambda\tilde{r}\right) + K_2\!\left(\lambda\tilde{r}\right)\Big], \tag{12}$$

substitution into (2) yields $\kappa_{\text{exact}}(\tilde{r})$, and the dimensional pressure jump is

$$\Delta P_{\text{exact}}(\tilde{r}) \;=\; \frac{\gamma}{R}\,\kappa_{\text{exact}}(\tilde{r}). \tag{13}$$
